# *Klebsiella pneumoniae*—A Useful Pathogenic Strain for Biotechnological Purposes: Diols Biosynthesis under Controlled and Uncontrolled pH Levels

**DOI:** 10.3390/pathogens8040293

**Published:** 2019-12-11

**Authors:** Laura Mitrea, Dan Cristian Vodnar

**Affiliations:** Faculty of Food Science and Technology, Institute of Life Sciences, University of Agricultural Sciences and Veterinary Medicine, Calea Mănăștur 3–5, 400372 Cluj-Napoca, Romania

**Keywords:** *Klebsiella pneumoniae*, pathogenic strain, pH, 1,3-propanediol, 2,3-butanediol, batch fermentation, viability

## Abstract

Despite being a well-known human pathogen, *Klebsiella pneumoniae* plays a significant role in the biotechnology field, being considered as a microbial cell factory in terms of valuable chemical biosynthesis. In this work, *Klebsiella pneumoniae* DSMZ 2026 was investigated for its potential to biosynthesize 1,3-propanediol (PDO) and 2,3-butanediol (BDO) during batch fermentation under controlled and uncontrolled pH levels. The bacterial strain was cultivated at a bioreactor level, and it was inoculated in 2 L of specific mineral broth containing 50 g/L of glycerol as the main carbon source. The process was conducted under anaerobic conditions at 37 °C and 180 RPM (rotations per minute) for 24 h. The effect of pH oscillation on the biosynthesis of PDO and BDO was investigated. Samples were taken every 3 h and specific tests were performed: pH measurement, main substrate consumption, PDO and BDO production. The cell morphology was analyzed on both solid and liquid media. After 24 h of cultivation, the maximum concentrations of PDO and BDO were 28.63 ± 2.20 g/L and 18.10 ± 1.10 g/L when the pH value was maintained at 7. Decreased concentrations of PDO and BDO were achieved (11.08 ± 0.14 g/L and 7.35 ± 0.00 g/L, respectively) when the pH level was not maintained at constant values. Moreover, it was identified the presence of other metabolites (lactic, citric, and succinic acids) in the cultivation media at the beginning of the process, after 12 h and 24 h of cultivation.

## 1. Introduction

Among Enterobacteriaceae, *Klebsiella* spp. are recognized mostly in the medical field as famous opportunistic germs associated with pathogenic infections [1,2]. *K. pneumoniae* represents a saprophytic pathogen that might affect both plants, animals, and humans [1,3,4], but it can be successfully used for biotechnological applications [5]. In medical terms, *K. pneumoniae* is a pathogenic strain responsible for multiple nosocomial infections, including pneumonia, urinary tract, and soft tissue infections, and septicemias [6,7,8]. *K. pneumoniae* cells are able to spread very quickly especially in a hospital environment, the main cause being the unclean hands of personnel [1,6]. The pathogenic potential of the Gram-negative *K. pneumoniae* is mainly due to the external cell structure. The main virulence factor is the outer cell membrane that consists of a capsule, lipopolysaccharides (recognized as endotoxins in humans), siderophores, and pili. The capsular polysaccharides are acidic structures attached to the peripheral membrane, which play a major role in capsular antigen synthesis and export [9,10,11,12]. In many cases, the capsule is also responsible for biofilm and mucus formation, a very important aspect of the infection settlement [2,10,13,14].

In the biotechnology field, pathogenic bacteria like *Klebsiella* (e.g., *K. pneumoniae*, *K. oxytoca*, *K. variicola*), *Clostridium* (e.g., *C. butyricum*, *C. acetobutylicum*, *C. pasteurianum*, *C. diolis*, *C. perfingens*), *Escherichia* (e.g., *E. coli*) or *Bacillus* (e.g., *B. cereus*, *B. subtilis*) have been studied for more than 100 years to produce important chemical compounds such as diols (1,2-propanediol, 1,3-propanediol, 1,4-butanediol, 2,3-butanediol), organic acids (lactic, acetic, citric, succinic, pyruvic) or alcohols (ethanol, 1-butanol) [15,16,17,18,19,20,21,22,23,24,25,26,27]. For biotechnological approaches and to scale-up the processes at an industrial level in safe conditions, many of the above-mentioned pathogenic strains have been genetically modified for the removal of the virulence factors [20,28,29,30]. In many cases, when pathogenic strains are used for a biotechnological application, specific safety protocols are followed and sterilization steps are involved [31]. Anyway, *K. pneumoniae* is one of the representative strains used at a large scale for the biosynthesis of PDO and BDO by means of fermentation processes, under both aerobic and anaerobic conditions, starting from glycerol as the main nutrient substrate [15,20,32]. The pathogenic strain of *K. pneumoniae* is mostly used for PDO biosynthesis at a large scale because of its natural ability to produce the B_12_ co-enzyme, which is a very important factor for the microbial synthesis of PDO and BDO [17,18,33]. Both PDO and BDO are platform chemical products with industrial applications, like biopolymers, solvents, and fuels [5,20,34,35]. PDO plays an important role in the synthesis of biodegradable plastics, namely for the polytrimethylene-terephthalate (PTT) production [36,37], while BDO appears as a by-product during the biosynthesis of PDO and one of its significant roles is in the production of synthetic rubber [18,20].

Based on our previous publications [17,33], the bacterial strain of *K. pneumoniae* DSMZ 2026 was chosen for pure glycerol fermentation in order to obtain PDO and BDO. In this study, *K. pneumoniae* was utilized in a bioreactor batch cultivation under anaerobic conditions, for the production of PDO and BDO. The fermentation process was conducted for 24 h, and the cell viability evolution, pH oscillation, PDO and BDO biosynthesis, other metabolites formation (lactic, citric, and succinic acids), and the substrate consumption were monitored. Moreover, the bacterial morphology of *K. pneumoniae* DSMZ 2026 on both solid and liquid media was analyzed.

## 2. Results and Discussion

The viability evolution, pH oscillation, glycerol consumption, PDO, BDO, and other metabolites (lactic, citric, and succinic acids) biosynthesis were monitored during 24 h of batch fermentation, and the results are presented in Table 1, Table 2 and Table 3. For the first trial where pH was maintained at constant values, the maximum concentration of PDO was registered after 18 h of cultivation (30.63 ± 1.99 g/L), while the maximum concentration for BDO was obtained after 24 h (18.10 ± 1.10 g/L) (Table 1). In the case of the batch fermentation where pH was not controlled, maximum metabolites concentration was recorded after 18 h of fermentation for PDO (12.45 ± 0.04 g/L) and BDO (9.75 ± 0.14 g/L). Considering the organic acids biosynthesis (Table 3), it was observed that lactic acid was present at the beginning of the process but it decreased after 12 h, while citric and succinic acids were identified after 12 h and 24 h of cultivation for both trials. According to Table 2, the PDO formation yield in case of the controlled pH trial, the results (0.68 mol_PDO_/mol_Glycerol_) were closed to the theoretical values (0.72 mol_PDO_/mol_Glycerol_) mentioned by scientific literature [17]. When pH was maintained constant, the yields obtained for BDO and other metabolites (organic acids) were significantly under the values of the theoretical yields. In the case of the trial with uncontrolled pH, the yields of PDO, BDO, lactic acid, citric acid, and succinic acid were considerably lower compared to the theoretical yields (Table 2).

Some authors suggested that the PDO production by *K. pneumoniae* cells was related to neutral pH values, while the BDO biosynthesis was related to lower pH values [38,39]. Moreover, the literature points out that the higher quantities of PDO were produced under anaerobic circumstances, while the aerobic conditions facilitated the BDO biosynthesis as the main product [19,40,41]. The higher concentrations of PDO presented in Table 1 compared with those obtained for BDO in both trials (controlled and uncontrolled pH) might be due only to the anaerobic conditions. In *Klebsiella* cells, glycerol was metabolized to PDO by the dehydratase system, which was activated under anaerobic conditions [41]. Considering the BDO production from glycerol, the aeration and pH levels were reported to be effective in the case of *Klebsiella* strains [19].

**Figure 1:**
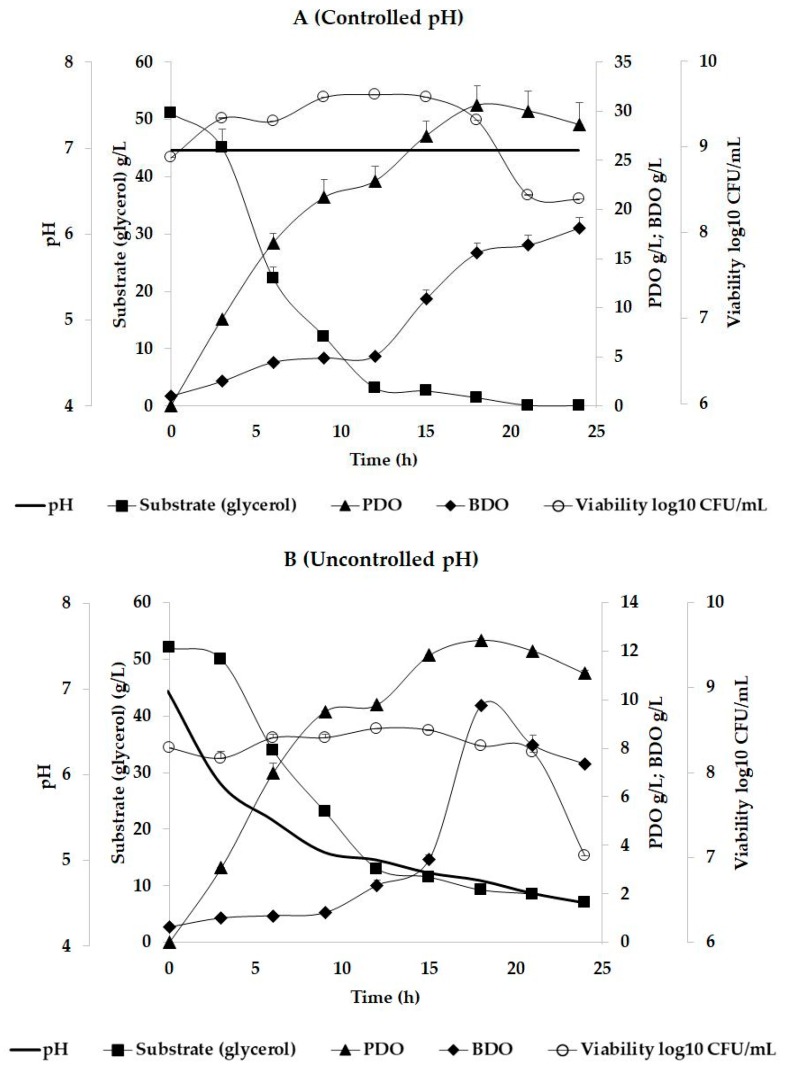
Batch cultivation of *K. pneumoniae* DSMZ 2026 into a 5 L bioreactor under (**A**) controlled and (**B**) uncontrolled pH.

For both trials with controlled and uncontrolled pH, the substrate was consumed almost entirely after 24 h of cultivation (Figure 1). The pH level influenced the metabolism of the *Klebsiella* cells by slowing down the assimilation of the substrate and metabolites biosynthesis. The pH decrease affected biomass formation through viability diminution. As previous studies reported [17,46,47], the optimal pH conditions for the microbial growth in case the of *Klebsiella* strains, were ranging between 6 and 8. A higher pH tolerance (e.g., 8) was related to the genetically modified strains, which were optimized for an elevated metabolite production such as PDO [47], while a lower pH level inhibits cell division [46]. 

PDO is a three-carbon diol with an important contribution in ecological materials division (e.g., biopolymers, polyesters, composites, coatings), while BDO is a four-carbon diol with a major role in the industry of polymers [34,35,48]. Considering the results obtained for PDO and BDO (Table 1, Figure 1), these were similar to those reported in the literature. For example, Cheng et al. [49] employed a *K. pneumoniae* strain M5al in batch cultivation at a bioreactor level and achieved 18 g/L PDO and up to 5 g/L of BDO after 18 h of cultivation, while the pH was maintained at 6.8 through the automatic addition of NaOH, and the starting substrate concentration was 40 g/L of glycerol. Da Silva et al. [46] obtained similar results on *K. pneumoniae* strain GLC29 in batch trials where pH values were maintained between 6.9 and 7.1. They achieved a final PDO concentration of 20 g/L after 9 h of fermentation and 1 g/L of BDO, starting from an initial glycerol concentration of 40 g/L. Kumar et al., [40] tested a mutant strain of *K. pneumoniae* J2B at a shake-flask level under different aeration circumstances. After 12 h of fermentation, they achieved a maximum concentration of 8 g/L of PDO under anaerobic conditions at a pH value of 5.6 and a starting glycerol concentration of 20 g/L. For the same study, the BDO synthesis was not initiated under the mentioned conditions [40]. Higher PDO concentrations for batch trials were obtained by Zhao et al. [50], who used microencapsulated *K. pneumoniae* type ZJU 5205. They achieved 63 g/L of PDO after 11 h of cultivation starting from a high initial glycerol concentration of 120 g/L [50]. Impressive amounts of BDO were reported by Durgapal et al. [42], who obtained 26.6 g/L by using *K. pneumoniae* J2B in a 48 h fed-batch cultivation under increased aeration rate and pH values less than 5. The same study reported a high concentration of PDO of about 58 g/L at constant values of pH at 7 [42].

**Figure 2:**
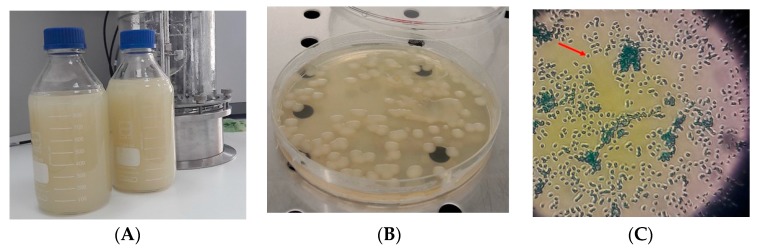
The *K. pneumoniae* DSMZ 2026 appearance after 24 h of cultivation on liquid media (**A**), Columbia solid media (**B**), and under microscopic examination after methylene blue staining (**C**). The red arrow indicates the capsule of the cell.

In order to observe the morphological characteristics, the *Klebsiella* strain was cultivated on Columbia agar plates and observed under an optical microscope. Placed on solid media and incubated at 37 °C for 24 h, *Klebsiella* cells developed large (>1 mm), opaque, cream-colored and glistening mucoid colonies (Figure 2B). Under microscope light, individual cells surrounded by a thin halo could be observed (Figure 2C), a structure that constitutes the capsule of the bacteria. According to Evrad et al. [51], the voluminous capsular layer was made of polysaccharides that cover the entire bacterial surface, and its role was to protect de bacteria cell against macrophage phagocytosis in animal and human models [51,52]. Actually, the thin halo observed under microscopic examination represented the capsular layer that gives the pathogenic features to the *Klebsiella* cells. In biotechnological processes instead, large amounts of capsular polysaccharides induce mucoviscosity [52] and obstruct the separation of bacteria cells from fermentation media during the downstream process. The capsular layer might influence the metabolites excretion in the fermentation medium [51,53]. In our study, the capsule formation did not significantly influence the accumulation of the metabolite in the cultivation broth, but the pH level was a limiting factor for metabolite production.

## 3. Materials and Methods

### 3.1. Microorganism and Culture Conditions

In this study, *K. pneumoniae* DSMZ 2026 obtained from the German Collection of Microorganisms and Cell Culture (DSMZ, Braunschweig, Germany) was used for two experiments: One experiment involved the control of pH during the cultivation, while in the other experiment the pH was not controlled. The cultivation conditions and fermentation broth components were similar to those reported by Menzel et al. [54]. The freeze-dried bacterial strain was activated on Columbia agar plates (peptone special, 23 g/L; starch, 1 g/L; NaCl, 5 g/L; agar, 15 g/L; pH 7.3 ± 0.2) at 37 °C for 24 h, and stored at 4 °C for 3 months.

The bacterial strain was pre-cultured in a mineral broth with the following composition: Glycerol 50 g/L; K_2_HPO_4_ 3.4 g/L; KH_2_PO_4_ 1.3 g/L; (NH_4_)_2_SO_4_ 2.0 g/L; MgSO_4_ × 7 H_2_O 0.2 g/L; yeast extract 2.0 g/L; CaCO_3_ 2.0 g/L; FeSO_4_ × 7 H_2_O 5.0 mg/L; CaCl_2_ 2.0 mg/L; ZnCl_2_ 0.14 mg/L; MnCl_2_ × 4 H_2_O 0.2 mg/L; H_3_BO_3_ 0.12 mg/L; CoCl_2_ × 6 H_2_O 0.4 mg/L; CuCl_2_ × 2 H_2_O 0.04 mg/L; NiCl_2_ × 6 H_2_O 0.05 mg/L; Na_2_MoO_4_ × 2 H_2_O 0.07 mg/L. After pre-culture, the bacterial strain was transferred in a bioreactor batch culture containing: Glycerol 50 g/L, KCl 0.75 g/L, NaH_2_PO_4_ × H_2_O 1.38 g/L, NH_4_Cl 5.35 g/L, Na_2_SO_4_ 0.28 g/L, MgCl_2_ × 6 H_2_O 0.26 g/L, CaCl_2_ × 2 H_2_O 0.29 g/L, citric acid × H_2_O 0.42 g/L, yeast extract 2.0 g/L, ZnCl_2_ × 6 H_2_O 3.4 mg/L, FeCl_2_ × 6 H_2_O 27 mg/L, MnCl_2_ × 4 H_2_O 10 mg/L, CuCl_2_ × 2 H_2_O 0.85 mg/L, CoCl_2_ × 6 H_2_O 2.35 mg/L, H_3_BO_3_ 0.5 mg/L, Na_2_MoO_4_ × 2 H_2_O 25 µg/L. All the reagents were of analytical grade, and the pH of the culture broth was adjusted to 7 by adding a few drops of 2 M NaOH before sterilization at 121 °C for 15 min.

### 3.2. Batch Cultivation at Bioreactor Level

**Figure 3:**
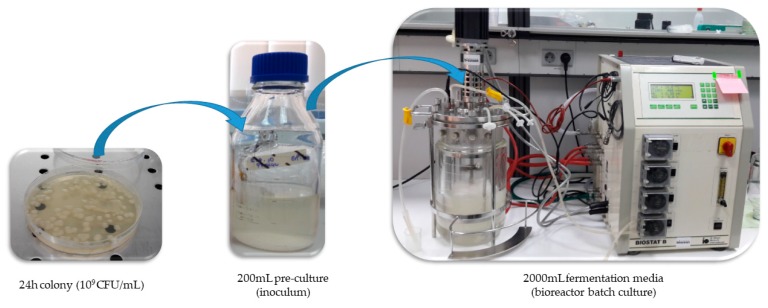
The batch fermentation steps.

Initially, 200 mL of pre-culture broth was inoculated with a 24 h colony of 10^9^ CFU/mL, as is shown in Figure 3. The pre-culture was maintained in anaerobic conditions at 37 °C for 24 h and 180 RPM. The experiment was performed by using a 5 L bioreactor (B. Braun Biotech International, Melsungen, Germany) filled with 2 L of culture broth bubbled with CO_2_ before the inoculation in order to remove the oxygen. The inoculum was added into the fermentation media under sterile conditions. The fermenter was fitted with temperature, pH, and rotation speed control. The temperature was maintained at 37 °C and rotations were maintained at 180 RPM for 24 h of cultivation. For one trial the pH was kept constant at 7 by the addition of NaOH 2 M, while for the other one the pH was not maintained constant in order to observe its oscillation influence over the cell viability, PDO, BDO, and organic acids (lactic, citric and succinic acids) biosynthesis. Samples were collected every 3 h during the cultivation for specific tests.

### 3.3. Testing Methods

Glycerol consumption, PDO, and BDO production were measured by HPLC after the derivatization process [55,56]. The HPLC unit (Agilent 1200, Santa Clara, CA, USA) was equipped with quaternary pump, solvent degasser, auto-sampler, UV-Vis photodiode detector (DAD) coupled with single quadrupole mass detector (MS, Agilent 6110), equipped with electrospray ionization source (ESI) (Agilent Technologies, Santa Clara, CA, USA), and controlled by Agilent ChemStation software. The ESI detection was performed by the following the work conditions: Capillary voltage 3100 V, 350 °C, nitrogen flow 7 L/min, m/z 100–500 full-scan. The compounds separation was done with an Eclipse XDB C18 column (5 μm, 4.6 × 150 mm I.D.) (Agilent Technologies) using the 20 mM NH_4_HCO_2_ mobile phase (A), pH 2.8, and (B) ACN/A (90/10, v/v) at a flow rate of 0.3 mL/min at 25 °C.

Lactic, citric, and succinic acids were determined using HPLC (Agilent 1200, Santa Clara, CA, USA) with an Aclaim OA (5 μm, 4 × 150 mm, Dionex, Waltham, MA, USA) reversed-phase chromatographic column coupled with a UVdetector, solvent degasser, quaternary pumps, column thermostat, and manual injector (Agilent Technologies). The chromatographic column was eluted for 10 min with 50 mM NaH_2_PO_4_, pH 2.8, with a flow rate of 0.5 mL/min, at 20 °C. The chromatograms were measured at 210 nm.

The viability of the *Klebsiella* cells was determined by plate counting method proposed by Ziegler and Halvorson [24,56,57]. Microscopic analysis consisting of methylene blue staining was applied in order to observe the bacterial cell morphology and appearance. A loop of fermentation sample collected after 24 h of cultivation was stained with 1mL of methylene blue, dried for 5 min, and examined under the optical microscope light at 400× magnification [58]. The model of the optical microscope used for our experiments was IOR ML-4M.

## 4. Conclusions

The bacterial strain of *K. pneumoniae* DSMZ 2026 was used in a batch cultivation process at the bioreactor level to biosynthesize PDO and BDO. *K. pneumoniae* grew successfully in mineral broth under an anaerobic environment, but metabolites biosynthesis was influenced by the pH alteration. *K. pneumoniae* was cultivated in 2 L of fermentation broth containing 50 g/L glycerol as the main substrate and maintained at 37 °C for 24 h at 180 RPM. After 24 h of cultivation was obtained 28.63 ± 2.20 g/L PDO and 18.10 ± 1.10 g/L BDO when the pH level was maintained at constant values. When the pH values decreased to 4.51, the concentrations obtained were only 11.08 ± 0.14 g/L PDO and 7.35 ± 0.00 g/L BDO. On solid media after 24 h of incubation at 37 °C, *K. pneumoniae* developed large, opaque, cream-colored, and glistening mucoid colonies. Under the microscope examination, there were observed individual cells surrounded by light halo constituting the bacterial capsular layer. The capsule formation did not significantly influence the accumulation of the metabolites in the cultivation broth, but the pH level was a limiting factor for the production of the metabolites in *K. pneumoniae* DSMZ 2026.

## Figures and Tables

**Table 1:** Results obtained after the cultivation of *K. pneumoniae* strain DSMZ 2026 under controlled and uncontrolled pH.

**Time (h)**	**pH**	**Substrate (glycerol) g/L**	**PDO g/L**	**BDO g/L**	**Viability log10 CFU/mL**
0	7.00	51.12 ± 0.02	0.00 ± 0.00	1.01 ± 0.00	8.91 ± 0.00
3	7.00	45.11 ± 3.21	8.86 ± 0.00	2.50 ± 0.00	9.37 ± 0.00
6	7.00	22.32 ± 2.00	16.59 ± 1.00	4.44 ± 0.02	9.33 ± 0.00
9	7.00	12.14 ± 0.98	21.22 ± 1.85	4.89 ± 0.09	9.61 ± 0.00
12	7.00	3.08 ± 0.02	22.89 ± 1.52	5.11 ± 0.10	9.64 ± 0.00
15	7.00	2.60 ± 0.19	27.51 ± 1.45	10.88 ± 0.92	9.62 ± 0.00
18	7.00	1.40 ± 0.02	30.63 ± 1.99	15.55 ± 1.03	9.34 ± 0.00
21	7.00	0.08 ± 0.00	30.03 ± 2.00	16.36 ± 1.00	8.47 ± 0.00
24	7.00	0.04 ± 0.00	28.63 ± 2.20	18.10 ± 1.10	8.42 ± 0.00
**Time (h)**	**pH**	**Substrate (glycerol) g/L**	**PDO g/L**	**BDO g/L**	**Viability log10 CFU/mL**
0	6.98	52.01 ± 0.00	0.00 ± 0.00	0.62 ± 0.00	8.30 ± 0.00
3	5.91	50.00 ± 0.00	3.08 ± 0.00	1.00 ± 0.00	8.18 ± 0.08
6	5.48	33.85 ± 0.00	7.00 ± 0.39	1.09 ± 0.00	8.42 ± 0.04
9	5.10	23.00 ± 0.10	9.52 ± 0.01	1.22 ± 0.00	8.42 ± 0.04
12	5.01	12.98 ± 0.58	9.80 ± 0.09	2.35 ± 0.21	8.53 ± 0.00
15	4.86	11.52 ± 0.88	11.83 ± 0.00	3.40 ± 0.21	8.51 ± 0.00
18	4.77	9.23 ± 0.07	12.45 ± 0.04	9.75 ± 0.14	8.33 ± 0.00
21	4.62	8.49 ± 0.04	12.01 ± 0.04	8.12 ± 0.43	8.26 ± 0.00
24	4.51	6.99 ± 0.06	11.08 ± 0.14	7.35 ± 0.00	7.04 ± 0.00

The shown data represent the mean values of three biological replicates, and the standard deviation (SD ±) is under 10%.

**Table 2:** Metabolite formation yield after 24 h of cultivation of *Klebsiella pneumoniae* DSMZ 2026 on glycerol.

Yield mol_Product_/mol_Substrate_	PDO	BDO	Lactic Acid	Citric Acid	Succinic Acid	References
Maximum theoretical yield	0.72	0.63	0.73	0.51	0.50	[17,41,42,43,44,45]
Controlled pH	0.68	0.35	0.00	0.02	0.03	This study
Uncontrolled pH	0.25	0.14	0.00	0.01	0.03	This study

**Table 3:** Identification of other metabolites in the cultivation broth after 12 h and 24 h.

**Controlled pH**				
**Time (h)**	**pH**	**Lactic Acid g/L**	**Citric Acid g/L**	**Succinic Acid g/L**
0	7.00	0.02 ± 0.00	0.00 ± 0.00	0.04 ± 0.00
12	7.00	0.00 ± 0.00	2.63 ± 0.02	1.96 ± 0.05
24	7.00	0.00 ± 0.00	2.67 ± 0.04	2.09 ± 0.05
**Uncontrolled pH**				
**Time (h)**	**pH**	**Lactic Acid g/L**	**Citric Acid g/L**	**Succinic Acid g/L**
0	6.98	0.02 ± 0.00	0.00 ± 0.00	0.16 ± 0.00
12	5.01	0.00 ± 0.00	1.11 ± 0.00	2.18 ± 0.02
24	4.51	0.00 ± 0.00	2.23 ± 0.052	2.35 ± 0.07

Data are average values and standard deviations (SD ± less than 10%) of triplicate experiments.
